# *Asparagus cochinchinensis *stimulates release of nerve growth factor and abrogates oxidative stress in the Tg2576 model for Alzheimer’s disease

**DOI:** 10.1186/s12906-017-1775-3

**Published:** 2018-04-06

**Authors:** Hyun Ah Lee, Ji Eun Kim, Ji Eun Sung, Woo Bin Yun, Dong Seob Kim, Hee Seob Lee, Jin Tae Hong, Dae Youn Hwang

**Affiliations:** 1 Department of Biomaterial Science, College of Natural Resources and Life Science/Life and Industry Convergence Research Institute, Pusan National University, Miryang, 627-706 South Korea; 2 College of Human Ecology, Pusan National University, Busan, 609-735 South Korea; 3 College of Pharmacy, Chungbuk National University, Chungju, 361-763 South Korea

**Keywords:** *Asparagus cochinchinesis*, Alzheimer’s disease, NGF, Anti-oxidant, Tg2576 mice

## Abstract

**Backgroud:**

Use of multifunctional drugs with neurotrophic supporting and oxidative stress suppressing activity may be considered a therapeutic strategy to protect or repair cellular damage caused during the progression of Alzheimer’s disease (AD). In this study, we investigated the therapeutic effects of aqueous extract of *A. cochinchinesis* root (AEAC), particularly its role as a nerve growth factor (NGF) stimulator and anti-oxidant in Tg2576 mice showing AD phenotypes of human.

**Methods:**

Tg2576 mice were received 100 mg/kg/day AEAC via oral administration, while mice in the Vehicle treated group received dH_2_O for 4 weeks. Non-Tg littermates were used as a control group. Following AEAC treatment for 4 weeks, NGF function, anti-oxidantive status, Aβ-42 peptide level, γ-secretase expression and neuronal cell functions were analyzed in the brain of Tg2576 mice.

**Results:**

AEAC containing flavonoids, phenols, saponins and protodioscin induced enhancement of NGF secretion and decreased intracellular ROS in the neuronal and microglial cell line. These effects as well as enhanced SOD levels were also detected in AEAC treated Tg2576 mice. The expression of p-Akt among downstream effectors of the high affinity NGF receptor was dramatically recovered in AEAC treated Tg2576 mice, while the expression of p75^NTR^ was slightly recovered in the same group. Significant recovery on the level of Aβ-42 peptides and the expression of γ-secretase members including PS-2, APH-1 and NCT were detected in AEAC treated Tg2576 mice. Furthermore, AEAC treated Tg2576 mice showed decreased numbers of dead cells and suppressed acetyl choline esterase (AChE) activity.

**Conclusions:**

These results suggest that AEAC contribute to improving the deposition of Aβ-42 peptides and neuronal cell injuries during the pathological progression stage of AD in the brain of Tg2576 mice through increased NGF secretion and suppressed oxidative stress.

## Background

AD is a well known neurodegenerative disorder characterized by the presence of senile plaques and neurofibrillary tangles in the hippocampus and the brain cortex that is accompanied by severe learning and memory impairment [1]. Senile plaques are mainly composed of Aβ peptides produced from the β-amyloid precursor protein (APP), while neurofibrillary tangles contain hyperphosphorylated and nitrated tau protein [2–4]. These two key components stimulate progression of the AD pathological process and accumulation of these abnormal proteins in specific regions of the brain [4].

Various attempts have reported several pathological mechanisms as the main cellular targets to prevent or retard the progression of AD to disease states [2]. Most approaches are designed to block or mitigate several pathological events, such as abnormal Aβ and tau aggregation, chronic inflammatory responses, and oxidative stress damage during the earliest stages [5–8]. Some other strategies stimulated the metabolism to decrease Alzheimer’s energetic failure and promote intrinsic mechanisms that protect or repair cellular damage, including synaptic plasticity, preservation of the lipid membrane composition, and promotion of damaged proteins and organelle turnover [9–11]. Furthermore, modulation of neurotransmission has been considered an additional therapeutic approach to avoid deficient cholinergic neurotransmission [12, 13]. Many recent studies have focused on multi-target therapies at early stages of the disease as the most effective strategy because of the complex conditions of the Alzheimer neurodegenerative process [2, 14].

Experimental therapeutic strategies of AD therapy have included supporting NGF and anti-oxidant treatment. Several herbal plants and medicinal foods exhibiting NGF stimulation activity have received increased attention as novel therapeutic strategies for the treatment of AD and its related diseases owing to limitations including transportation, uncertain effects, short half-life, poor safety and stability in the therapeutic application of exogenous NGF for AD [15, 16]. Glycyrrhizae radix and onjisaponins of *Polygala tenuifolia* increased NGF levels in rat embryonic astrocytes and PC12 cells [17, 18]. Also, the concentration of NGF in serum and brain tissue was significantly enhanced in AD models treated with ginsenoside Rb1 from *Panax quinquefolium* L. [19], red *Liriope platyphylla* [20, 21], fermented soybean products [22, 23], diosgenin [24] and propentofylline [25]. Furthermore, the possibility applying anti-oxidants to prevent and treat AD was supported by evidence that oxidative stress plays a functional role as a predecessor of other hall markers for neurodegeneration in the pathogenesis of AD [26, 27]. Some nutritional anti-oxidants including resveratrol [28], curcumin [29], epigallocatechin, gallate [30], L-acetyl-carnitine [31], vitamin E [32] and ascorbic acid [33] have shown potential beneficial effects as preventive drugs for AD. Despite above primary results about NGF and anti-oxidant, it has never been considered as a novel therapeutic strategy for multifunctional drug with enhancing NGF secretion and suppressing oxidative stress to treat AD.

Especially, several evidences as multifunctional drug of *A. cochinchinensis* for AD treatment had been provided by previous studies based on the therapeutic role of NGF stimulator and anti-oxidant, while *A. cochinchinensis* has been widely applied to improve the symptoms of several chronic diseases including inflammatory disease, breast cancer, brain disease, fever, cough and kidney disease [34–36]. The aqueous extract of *A. cochinchinensis* (AEAC) was increased in the concentration of the NGF protein in the supernatant of B35 cells and then secreted NGF induced dendritic outgrowth and downstream signaling pathway of NGF receptor in PC12 cells [37]. Also, this extract could obviously increase NOS, CAT, and SOD activities and the NO content, and reduce the MDA content in D-galactose induced mice aging model [38], while it showed high DPPH, nitrite and hydroxyl radical-scavenging activity with dose-dependent manner in vitro [39]. Furthermore, quercetin (AC01) isolated from the methanol extract of *A. cochinchinensis* had strong antioxidant activity with IC_50_ = 14.52 ± 2.12 μg/ml [40].

Therefore, in this study, we investigated the fundamental mechanisms responsible for NGF stimulation and anti-oxidative activities of AEAC in the Tg2576 model expressing human-like AD phenotypes to provide scientific evidence of its potential usefulness as a multiple target drug for treatment of neurodegenerative disorders.

## Methods

### Preparation of AEAC

AEAC were prepared as previously described [37]. Briefly, the roots of *A. cochinchinensis* harvested in the Gochang area were obtained from the Gochange National Agricultural Cooperation Federation (http://www.gochnh.com) in Korea. Root samples were dried in a drying machine (FD5510S-FD5520S, Ilshinbiobase Co., Dongducheon, Korea) at 60 °C and deposited as voucher specimens of *A. Cochinchinensis* roots (WPC-14-003) in the functional materials bank of the Wellbeing Regional Innovation System Center at Pusan National University (PNU). Samples were also identified by Dr. Shin Woo Cha at the Herbal Crop Research Division, National Institute of Horticultural & Herbal Science. Dry roots were reduced to powder using a pulverizer (MF-3100S, Hanil Electric Co., Seoul, Korea), after which AEAC was obtained at 121 °C for 45 min in a fixed liquor ratio (solid powder of *A. cochinchinensis*/dH_2_O ratio, 75 g: 500 ml) using circulating extraction equipment (Woori Science Instrument Co., Pocheon, Korea). The extract solutions were subsequently passed through a filter membrane (Whatman No. 2), after which they were concentrated by vacuum evaporation and lyophilization using circulating extraction equipment (IKA Labortechnik, Staufen, Germany). Finally, the AEAC powder was dissolved in dH_2_O or DMEM (Thermo Scientific, Waltham, MA, USA) to 1 mg/ml, then further diluted to the required concentration.

### Analysis of bioactive compounds in AEAC

The level of three bioactive compounds including total phenols, flavonoids and crudal saponins in AEAC were measured as previously described. The amount of total phenol in AEAC was determined according to the Folin-Ciocalteu method [41]. Briefly, the collected sample (20 μl) was mixed with 100 μL of 0.2 N Folin-Ciocalteu reagent for 5 min, after which 300 μl of 20% sodium carbonate was added. Following incubation at room temperature for 2 h the absorbance of the reaction mixture was measured at 765 nm. Gallic acid was used as a standard to produce the calibration curve. Total phenolic content was expressed in milligrams of gallic acid equivalents per gram of AEAC extract.

The amount of total flavonoids in AEAC was determined as described by Meda et al. [42]. Briefly, AEAC (200 μl) was added to test tubes containing 60 μl of 5% potassium nitrite, 600 μl of dH_2_O, and 60 μl of 10% aluminum chloride. After incubation at 25 °C for 5 min, the absorbance of the reaction mixture was measured at 510 nm. Total flavonoids content was determined using a standard curve with quercetin as a standard and expressed as milligrams of quercetin equivalents per gram of AEAC powder.

The total crude saponins were also detected as previously described [43]. Briefly, AEAC dissolved in 30 ml dH_2_O were repeatedly extracted with ethyl ether (50 ml) to remove the lipid soluble substances. After collection of the aqueous layer, they were further extracted with n-butanol (30 ml) three times. This layer was concentrated by vacuum evaporation and lyophilization using circulation extraction equipment (IKA Labortechnik). Finally, the total crude saponins was calculated using the following equation:

Crudal saponin (mg/g) = A-B/S

where, A is the dry weight of the n-butanol layer (mg), B is the weight of the flask (mg) and S is the solid volume of the sample (g).

### High performance liquid chromatography (HPLC) analysis

The protodioscin in AEAC was analyzed using an ILC 3000 HPLC system (Interface Engineering Co. Ltd., Seoul, Korea) equipped with a Corona® CAD® Detector (ESA Biosciences, Inc., Chelmsford, MA, USA). Chromatographic separation was performed on a CapCell PAK MG C18 (4.6 × 250 mm, particle size 5 μm; Shiseido Co., Ltd., Tokyo, Japan). The mobile phase consisted of solvent A (dH_2_O) and solvent B (acetonitrile) using the gradient elution program: 0–25 min, 30–90% solvent B and 25–40 min, 90% solvent B. A flow rate of 1.0 ml/min was used for sample analysis. The nebulizer gas was compressed nitrogen. The gas flow rate and gas pressure were maintained at 1.53 l/min and 35 ± 2 psi, respectively. The output signal of the detector was recorded using the Clarity™ Chromatography Software (DataApex, Prague, Czech Republic).

### Free radical scavenging activity

The scavenging activity of two, 2-diphenyl-1-picrylhydrazyl (DPPH) radical was measured as previously described [44]. Briefly, powdered extract was dissolved in 50% EtOH (100 μL) to give 13 different concentrations of AEACs (16 to 2,000 μg/ml), mixed with 100 μL of 0.1 mM DPPH (Sigma-Aldrich Co.) in 95% ethanol solution or 100 μL of 95% ethanol solution, then incubated at room temperature for 30 min. Next, the absorbance of the reaction mixture was measured at 517 nm using a Versa-max plate reader (Molecular Devices, Sunnyvale, CA, USA). The DPPH radical scavenging activity of the AEAC was expressed as the percent decrease in absorbance relative to the control. The IC_50_ value is defined as the concentration of substrate that causes a 50% loss in DPPH activity.

### In vitro analysis of neuronal cells

B35 cells, a neuroblastoma that originated from the central nervous system of rats (*Rattus norvegicus*), were seeded at a density of 5 × 10^4^ cells/0.2 ml and grown for 24 h in a 37 °C incubator. When the cells reached 70–80% confluence, they were either treated with vehicle (1× PBS) or pretreated with 100 μg/ml of AEAC dissolved in 1× PBS for 24 h. After collecting the supernatants, they were analyzed for NGF concentration and used for neuritic outgrowth analysis. The neuritic outgrowth of PC12 cells was measured as previously described [20]. Following incubation of PC12 cells treated with AEAC-conditioned medium for 24 h, the morphology of PC12 cells was observed under a microscope at 200× magnification (Leica Microsystems, Switzerland). The length of dendrites of PC12 cells was analyzed using the Leica Application Suite (Leica Microsystems, Switzerland).

Intracellular ROS levels in BV-2 cells, which are macrophage cells that originated from the Abelson murine leukemia virus-induced tumor, were measured by staining with 2′,7′-dichlorofluorescein diacetate (DCFH-DA) (Sigma-Aldrich Co.). Briefly, BV-2 cells were seeded at 5 × 10^5^ cells/2 ml in 6-well plates, then grown with 100 μg/ml of AEAC for 1 h in a 37 °C incubator. After washing once with 1× PBS, the cells were incubated with 0.5 μg/ml of LPS (Sigma-Aldrich Co.) for another 24 h. Next, cells were incubated with 25 μM DCFH-DA for 30 min at 37 °C. Finally, the cells were washed twice with PBS, after which the green fluorescence was observed at 200× and 400× magnification using a fluorescent microscope (Eclipse TX100, Nikon, Tokyo, Japan).

### Experimental design for animals

The animal protocol used in this study was reviewed and approved by the Pusan National University-Institutional Animal Care and Use Committee (PNU-IACUC; Approval Number PNU-2015-0991). Adult Tg2576 mice and Non-Tg mice were purchased from Samtaco (Osan, Korea) and handled at the Pusan National University Laboratory Animal Resources Center, which is accredited by the Korea Food and Drug Administration (FDA) (Accredited Unit Number-000231) and AAALAC International (Accredited Unit Number; 001525). All mice were provided with a standard irradiated chow diet (Purina Mills Inc., Seoungnam, Korea) *ad libitum*, and were maintained in a specific pathogen-free state under a strict light cycle (lights on at 08:00 h and off at 20:00 h) at 23 ± 2 °C and a relative humidity of 50 ± 10%.

Tg2576 mice (*n* = 13, 12–24 months old) were assigned to either a vehicle treated group or an AEAC treated group (*n* = 6–7). Mice in the AEAC treated group were received 100 mg/kg/day AEAC via oral administration, while those in the vehicle treated group received dH_2_O for 4 weeks. Non-Tg littermates were used as a control group. Following AEAC treatment for 4 weeks, all animals were immediately sacrificed using CO_2_ gas, after which blood and tissue samples were collected and stored in Eppendorf tubes at −70 °C until assayed.

### Enzyme-linked immunosorbent assay (ELISA) for NGF

The concentrations of NGF in culture supernatant from B35 cells treated with AEAC as well as serum collected from the mice of subset group were measured using a NGF ELISA kit (Chemicon International Inc., Temecula, CA, USA). Briefly, samples and standards were incubated overnight on antibody-coated plates in a plate shaker at 100–150 rpm and 2–8 °C. Following the addition of anti-mouse NGF monoclonal antibody (100 μl), plates were subsequently incubated in a shaker for 2 h at room temperature, after which 100 μl of peroxidase conjugated donkey anti-mouse IgG polyclonal antibody was added to each well and samples were incubated at room temperature for an additional 2 h. After washing, 100 μl of TMB substrate was added to each well and the plate was incubated at room temperature for 15 min. The reaction was then quenched by the addition of 100 μl of stop solution, after which the plate was analyzed using a SoftMax Pro5 spectrophotometer (Molecular Devices, Sunnyvale, CA, USA).

### Western blot and slot blot

Total protein was prepared from the cortex and hippocampus tissue of mice in subset groups using Pro-Prep Protein Extraction Solution (iNtRON Biotechnology, Seongnam, Korea), then quantified using a SMARTTM BCA Protein Assay Kit (Thermo Scientific, Waltham, MA, USA) for Western blot analysis. Briefly, these proteins were separated by 4–20% sodium dodecyl sulfate-polyacrylamide gel electrophoresis (SDS-PAGE) for 2 h, after which resolved proteins were transferred to nitrocellulose membranes for 2 h at 40 V. Each membrane was then incubated separately at 4 °C with the following primary antibodies overnight: anti-TrkA antibody (Cell Signaling Technology, Beverley, MA, USA), anti-p-TrkA antibody (Cell Signaling Technology), anti-Akt antibody (Cell Signaling Technology), anti-p-Akt antibody (Cell Signaling Technology), anti-ERK antibody (Santa Cruz Biotechnology, Santa Cruz, CA, USA), anti-p-ERK antibody (Santa Cruz Biotechnology), anti-p75^NTR^ antibody (Cell Signaling Technology), anti-JNK antibody (Cell Signaling Technology), anti-p-JNK antibody (Cell Signaling Technology) and anti-actin antibody (Sigma-Aldrich Co.). Next, the membranes were washed with washing buffer (137 mM NaCl, 2.7 mM KCl, 10 mM Na_2_HPO_4_, and 0.05% Tween 20), then incubated with 1:1000 diluted horseradish peroxidase (HRP)-conjugated goat anti-rabbit IgG (Invitrogen, Carlsbad, CA, USA) at room temperature for 1 h. Finally, membrane blots were developed using Amersham ECL Select Western Blotting detection reagent (GE Healthcare, Little Chalfont, UK). The chemiluminescence signals that originated from specific bands were detected using FluorChemi®FC2 (Alpha Innotech Co., San Leandro, CA, USA).

To conduct slot blot analysis, 12.5 μg protein of hippocampus were transferred to a nitrocellulose membrane using a Slot Blot kit (Amersham Pharmacia Biotech, Piscataway, NJ, USA). The membrane was subsequently treated with primary rabbit anti-Aβ-42 peptide (Invitrogen, Carlsbad, CA, USA) antibody and horseradish peroxidase (HRP)-conjugated goat anti-rabbit IgG (Invitrogen). Finally, a signal on the membrane was detected by the same method used in western blot.

### Histological analysis

The perfusion and Nissl staining of the brain were performed as previously described [45]. Briefly, mice were anesthetized by intraperitoneal injection of Zoletile (150 mg/kg body weight), then transcardially perfused with 1× PBS followed by 4% formaldehyde to effectively remove blood and fix brain tissue. Following perfusion, each mouse brain was isolated from the skull and fixed overnight in formaldehyde, after which each brain was dehydrated and embedded in paraffin. Next, a series of brain sections (10 μm) were cut from paraffin-embedded tissue using a Leica microtome (Leica Microsystems, Bannockburn, IL, USA). For Nissl staining, these sections were deparaffinized with xylene, rehydrated with ethanol at graded decreasing concentrations of 100–70%, and finally washed with distilled water. The slides with brain sections were then subjected to Nissl staining solution with 0.1% cresyl violet acetate for 8 min, then washed with dH_2_O, after which the dead neuronal cells were enumerated by microscopic observation.

For immunohistochemical analysis of Aβ-42 peptides, the brain sections were deparaffinized with xylene, rehydrated, and pretreated for 30 min at room temperature with PBS blocking buffer containing 10% normal goat serum (Vector Laboratories Inc. Burlingame, CA, USA). The sections were then incubated with anti-Aβ-42 peptide antibody (Chemicon International, Inc. Billerica, MA, USA) at a dilution of 1:100 in tris-buffered saline (TBS) blocking buffer for 12 h. The antigen-antibody complexes were subsequently visualized with biotinylated secondary antibody (goat anti-mouse)-conjugated HRP streptavidin (Histostain-Plus Kit; Zymed, South San Francisco, CA, USA) at a dilution of 1:100 in TBS blocking buffer. Aβ peptides were detected using stable 3,3′-diaminobenzidine (DAB; Invitrogen, Carlsbad, CA, USA) and observed with Leica Application Suite (Leica Microsystems).

### Determination of AChE activity

The AChE assay was performed using an AChE assay kit (Abcam, Cambridge, UK) according to the manufacturer’s protocols. Briefly, the hippocampus from the brain of each mouse was homogenized in PRO-PREP protein extraction solution (1.0 mM PMSF, 1.0 mM EDTA, 1.0 μM pepstatin, 1.0 μM leupeptin, and 1.0 μM aprotinin, iNtRON Biotechnology, Inc., Seoul, Korea), after which the homogenates were stored at −70 °C until analysis. The samples or standards and ACh reaction mixtures were then incubated on a 96-well plate for 20 min at room temperature while protected from the light. Color alterations were read using a Vmax plate reader (Molecular Devices, Sunnyvale, CA, USA) at 405 nm.

### Determination of anti-oxidative activity

The total antioxidant capacity in serum samples was determined using a commercially available assay kit (ImAnOx TAS/TAC kit; Immundiagnostik, Stubenwald-Allee, Bensheim, Germany) according to the manufacturer’s instructions. Briefly, 10 μl of sample or calibrator was added to appropriate wells of a 96-well plate. Next, 100 μl of reagent 1 (reaction buffer A + peroxide solution) was added to each well and incubated for 10 min at 37 °C. Following this, 100 μl of reagent 2A (reaction buffer A + reaction buffer B + enzyme solution) and 100 μl of reagent 2B (reaction buffer A + reaction buffer B) were added to each well and incubated for 5 min at room temperature. Finally, stop solution was added to each well and the plate was read immediately using a SoftMax Pro5 spectrophotometer (Molecular Devices, Sunnyvale, CA, USA) at 450 nm. Quantification was performed using the calibrator data and the results were expressed as μmol of hydrogen peroxide (H_2_O_2_) equivalents per liter.

### Analysis of SOD activity

The SOD activity in the hippocampus tissue was detected using a calorimetric assay and the reagents in a SOD assay kit (Dojindo Molecular Technologies, Inc., Japan). First, these tissues (100 mg) was homogenized in 600 μl of sucrose buffer (0.25 M sucrose, 10 mM HEPES, 1 mM EDTA, pH 7.4) using a glass homogenizer. The lysate was then harvested from the mixture by centrifugation at 10,000 g for 60 min and stored at −70 °C until needed for the enzyme activity assay. To measure the SOD activity, the sample lysate was diluted with dilution buffer or saline as follows: 1, 1/5, 1/5^2^, 1/5^3^, 1/5^4^, 1/5^5^, 1/5^6^. Aliquots of the sample solution (25 μl) were subsequently placed in the wells of a 96-well plate, after which 200 μl of WST working solution was added. An enzyme working solution (20 μl) was also added to each sample well and mixed thoroughly. The enzyme reaction was then induced by incubating the mixture plate at 37 °C for 20 min, after which the absorbance was measured using a spectrophotometer at 450 nm. Finally, the SOD activity was calculated directly using the following equation:

SOD activity (inhibition rate %) =

[(A_blank1_-A_blank3_)-(A_sample_-A_blank2_)]/(A_blank1_-A_blank3_) × 100,

where, Ablank 1, 2, and 3 is the absorbance of blank 1, 2, and 3, respectively and Asample is the absorbance of the sample.

### Statistical analysis

Tukey’s post hoc test (SPSS for Windows, Release 10.10, Standard Version, Chicago, IL, USA) was used to identify significant differences between Non-Tg and Tg2576 groups, or the Vehicle treated Tg2576 and AEAC treated Tg2576 groups. All values are reported as the means ± standard deviation (SD) and a *p* value <0.05 was considered significant.

## Results

### Biochemical properties of AEAC

**Fig. 1:**
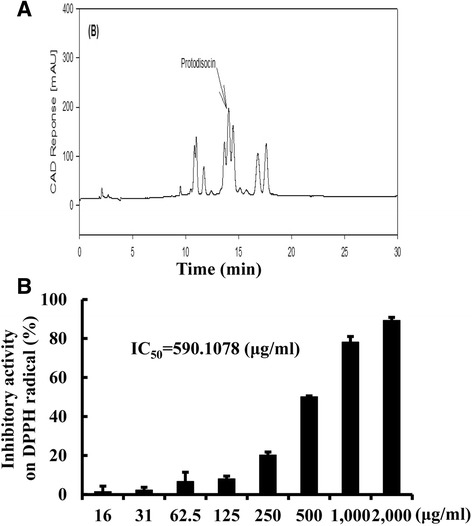
Biochemical properties of AEAC. **a** Chromatograms of the protodioscine were obtained by high performance liquid chromatography of AEAC. The peak height/area reflect the concentration of the protodioscine in AEAC. **b** Free radical scavenging activity of AEAC. DPPH radical scavenging activity was assayed in a mixture containing 0.1 mM DPPH and a range of concentrations of AEAC (16 – 2,000 μg/ml). DPPH, 2,2-diphenyl-1-picrylhydrazyl radical; IC_50_, half maximum inhibitory concentration. Values are presented as the means ± SD of three replicates

As shown in Table 1, AEAC contained 1.32 mg/g, 13.8 mg/g and 57.2 mg/g of total flavonoids, total phenol, and crudal saponins, respectively. Protodioscin was also detected as a sharp specific peak in the HPLC curve of AEAC at 14.1 min (Fig. 1a). Furthermore, the inhibitory activity against DPPH radical was gradually increased by the addition of 0.12–500 μg/ml of AEAC. Based on these data, the IC_50_ value of AEAC was determined to be 590.1 μg/ml (Fig. 1b). These results demonstrate that AEAC contained various bioactive components with anti-oxidant activity, and that these were likely related to its neuroprotective effects.

**Table 1:** The concentration of three bioactive compounds, total phenols, flavonoids and crudal saponins in AEAC

Category	Concentration (mg/g) in AEAC
Total flavonoids	1.32 ± 0.2
Total phenol	13.8 ± 0.9
Crudal saponin	57.2 ± 1.1

### In vitro efficacy of AEAC in neuronal cells

**Fig. 2:**
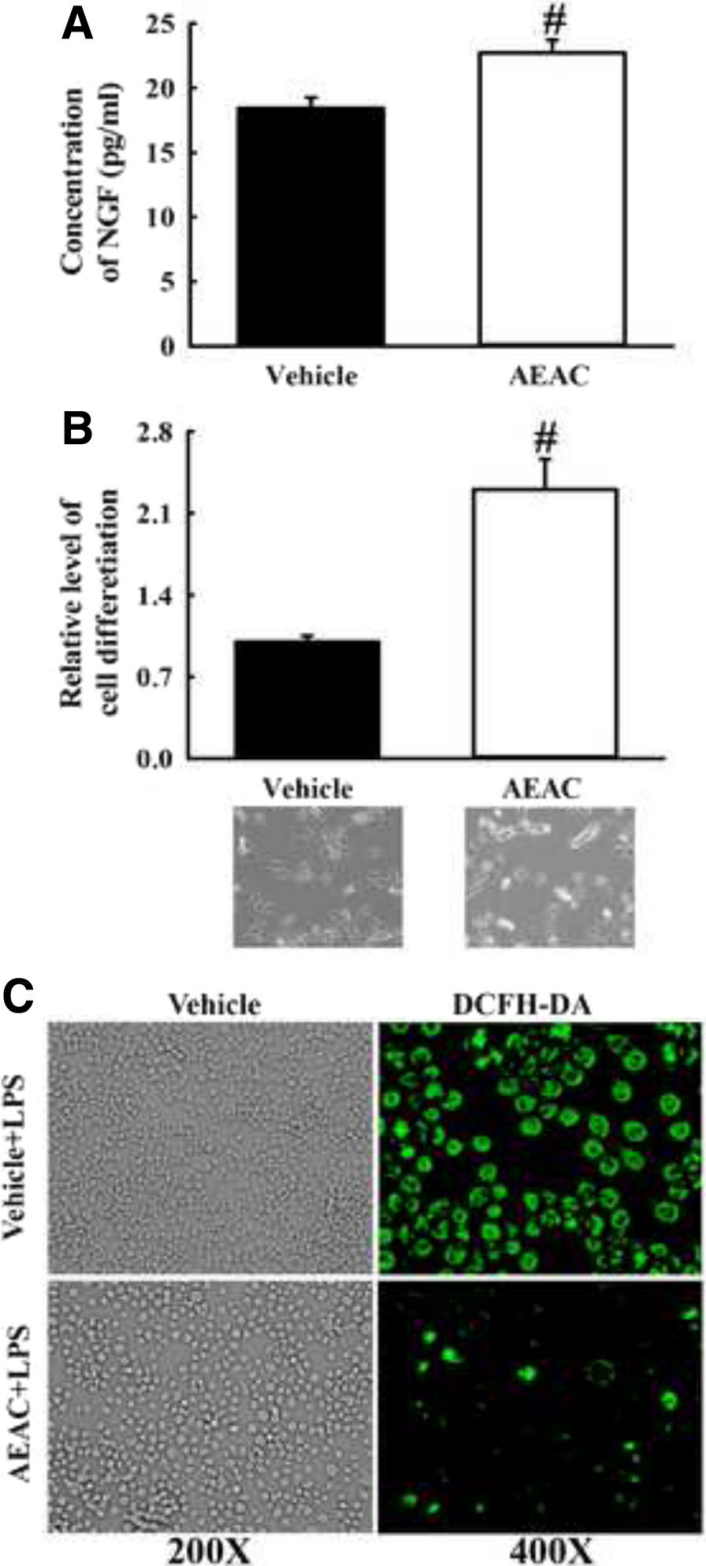
NGF stimulation capacity and anti-oxidant activity of AEAC in neuronal cells. **a** NGF concentration in the supernatant collected from each group was measured using an anti-NGF ELISA kit NGF. **b** Conditional medium (CM) was collected from B35 cells stimulated with AEAC for 24 h, then transferred to undifferentiated PC12 cells. After 24 h the cell morphology was observed at 200× using a microscope. The length of PC12 cells was measured using Leica Application Suite (Leica Microsystems). Values shown represent the means ± SD of three experiments. ^#^, *P* < 0.05 compared to the Vehicle treated group. **c** Determination of intracellular ROS production. After DCFH-DA treatment, green fluorescence in cells of subset groups was observed using a fluorescent microscope (Eclipse TX100, Nikon, Tokyo, Japan). BV-2 cells in each square of a 200× magnification image (left column) were further examined under 400× magnification (right column)

To examine the potential therapeutic effects of AEAC before of animal model study, alterations in the NGF secretion ability and anti-oxidative activity were measured in neural cell line treated with AEAC. The concentration of NGF protein increased significantly in the supernatant of B35 cells treated with AEAC compared with the Vehicle treated group (Fig. 2a). Moreover, the NGF in AEAC-conditioned media successfully induced the differentiation of undifferentiated PC12 cells. Dendrites were longer in the AEAC-conditioned medium cultured groups than the Vehicle-conditioned medium cultured group (Fig. 2b). Furthermore, a similar result was detected in the inhibitory effects of AEAC against LPS-induced ROS production. ROS production was dramatically decreased in AEAC+LPS treated BV-2 cells without any significant change on their morphology (Fig. 2c). Therefore, the above results from neuronal cells show the possibility that AEAC can successfully induce enhanced NGF secretion and suppress oxidative stress in an animal model for AD.

### Stimulatory effects of AEAC on NGF secretion in Tg2576 mice

**Fig. 3:**
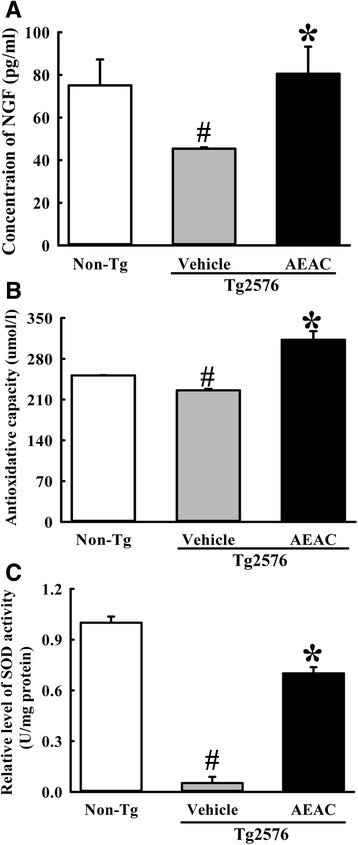
NGF stimulation capacity and anti-oxidant activity of AEAC in Tg2576 mice. **a** Detection of NGF secretion in Tg2576 mice. NGF concentration was measured in serum of subset groups using an anti-NGF ELISA kit. **b** The anti-oxidant capacity was determined in serum collected from mice using a lipid peroxidation assay kit that could detect MDA at 0.1 nmole/mg to 20 nmole/mg. **c** SOD activity of the hippocampus collected from mice using an SOD assay kit. One unit of SOD is defined as the amount of enzyme in 20 μl of sample solution that inhibits the reduction reaction of WST-1 with superoxide anion by 50%. Data represent the means ± SD of three replicates. ^#^, *p* < 0.05 compared to the Non-Tg group; *, *p* < 0.05 compared to the Vehicle treated Tg2576 group

To measure the effects of AEAC on the stimulation of NGF secretion in Tg2576 mice, the concentrations of NGF were measured in the blood serum after AEAC treatment for 4 weeks. The concentration of NGF was 44% lower in the Vehicle treated Tg group than the Non-Tg group. However, this level was significantly enhanced by 88% in the AEAC treated Tg group compared to the Vehicle treated Tg group and completely recovered to those of the Non-Tg group (Fig. 3a). These findings suggest that AEAC treatment can enhance NGF secretion in Tg2576 mice.

### Suppressive effects of AEAC treatment against Aβ-induced oxidative stress in Tg2576 mice

We also examined the suppressive effects of AEAC on oxidative stress induced by Aβ accumulation. To accomplish this, the anti-oxidative capacity and SOD activity were measured in the brains of Tg2576 mice. The antioxidative capacity in the brain tissue was 14.8% lower in the vehicle treated group than in the Non-Tg group. However, this level was significantly increased with 43.2% after AEAC treatment when compared with the Vehicle treated group (Fig. 3b). The activity of SOD was very similar to the antioxidant capacity. Specifically, the Vehicle treated group showed very lower SOD activity than the Non-Tg group, although this level was significantly higher in the AEAC treated groups (Fig. 3c). Overall, these results suggest that AEAC treatment inhibits the oxidative stress induced by Aβ peptide through enhancement of the antioxidative capacity and SOD activity.

### Effects of AEAC on NGF receptor signaling pathway

**Fig. 4:**
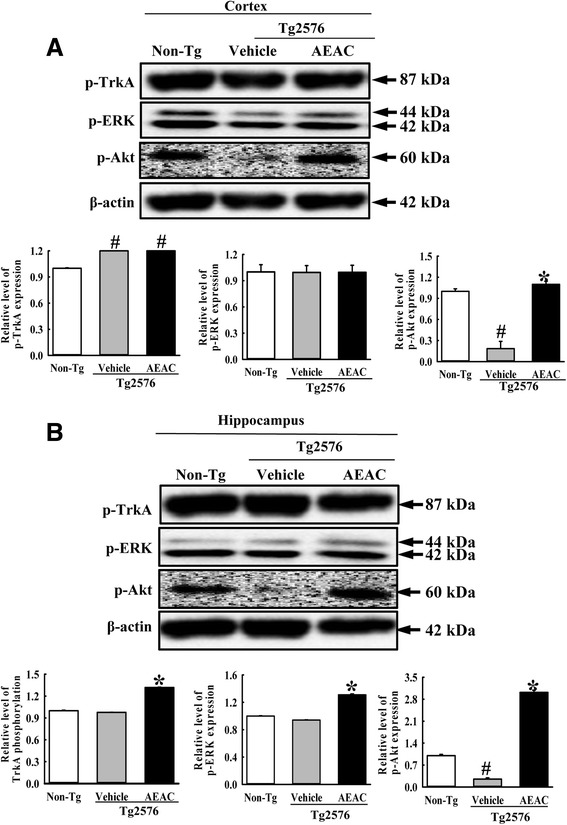
Downstream signaling pathway of TrkA NGF receptor. Tissue lysates were prepared from the cortex **a** and hippocampus **b** of Tg2576 mice treated with vehicle or AEAC as described in the Methods. Fifty micrograms of protein per sample was immunoblotted with antibodies for each protein. Three samples were assayed in triplicate by Western blotting. Data are reported as the means ± SD. ^#^, *P* < 0.05 compared to the Non-Tg group. *, *p* < 0.05 compared to the Vehicle treated Tg2576 group

NGF secreted from the brains of mice in the AEAC treated group transduce their signal into the cytosol by binding two types of NGF receptors located on the target cell’s membrane. Therefore, we investigated whether the enhancement of the NGF concentration induced by AEAC treatment could affect the two NGF receptor signaling pathways in the cortex and hippocampus of Tg mice. Analysis of the high affinity receptor revealed dramatic alterations in the expression level of p-Akt among three members. The p-Akt level was rapidly recovered in the cortex and hippocampus of Tg2576 mice by AEAC treatment for 4 weeks, although it was very lower in vehicle treated Tg2576 mice than Non-Tg mice (Fig. 4). Moreover, a slight recovery of the expression of p-ERK in the AEAC treated group was measured, while a constant level of p-TrkA expression was maintained (Fig. 4).

**Fig. 5:**
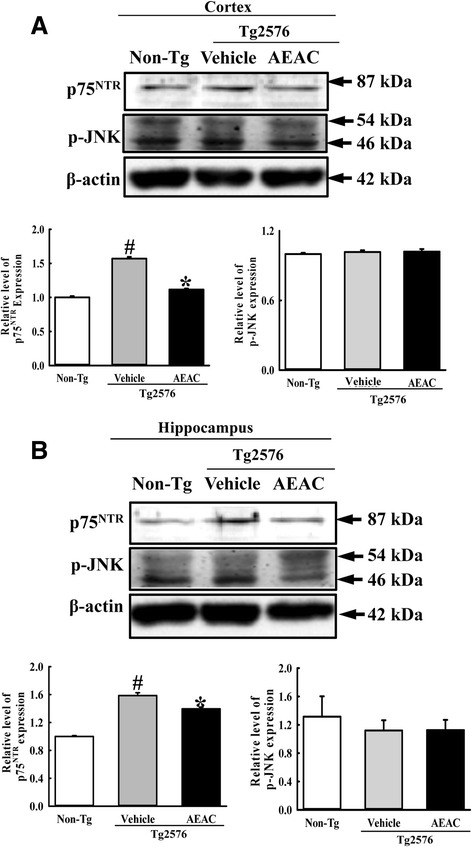
Downstream signaling pathway of p75^NTR^ NGF receptor. Tissue lysates were prepared from the cortex **a** and hippocampus **b** of Tg2576 mice treated with vehicle or AEAC as described in the Methods. Fifty micrograms of protein per sample were immunoblotted with antibodies for each protein. Three samples were assayed in triplicate by Western blotting. Data are reported as the means ± SD. ^#^, *P* < 0.05 compared to the Non-Tg group. *, *p* < 0.05 compared to the Vehicle treated Tg2576 group

Conversely, in the case of low affinity receptor, the expression level of p75^NTR^ was higher in Vehicle treated Tg2576 mice than Non-Tg mice. After AEAC treatment, this level was decreased in the cortex and hippocampus of AEAC treated Tg2576 mice relative to Vehicle treated Tg2576 mice (Fig. 5). However, the expression patterns of p-JNK, which is a downstream member of p75^NTR^, was not complete reflected to the expression level of p75^NTR^. The level of p-JNK expression was significantly decreased in only hippocampus of AEAC-treated Tg2576 mice (Fig. 5). Therefore, the above results showed that enhancement of the NGF level induced by AEAC treatment could induce significant changes in the NGF receptor TrkA and p75^NTR^ signaling pathways.

### Effects of AEAC on the production and deposition of Aβ peptides in the brain of Tg2576 mice

**Fig. 6:**
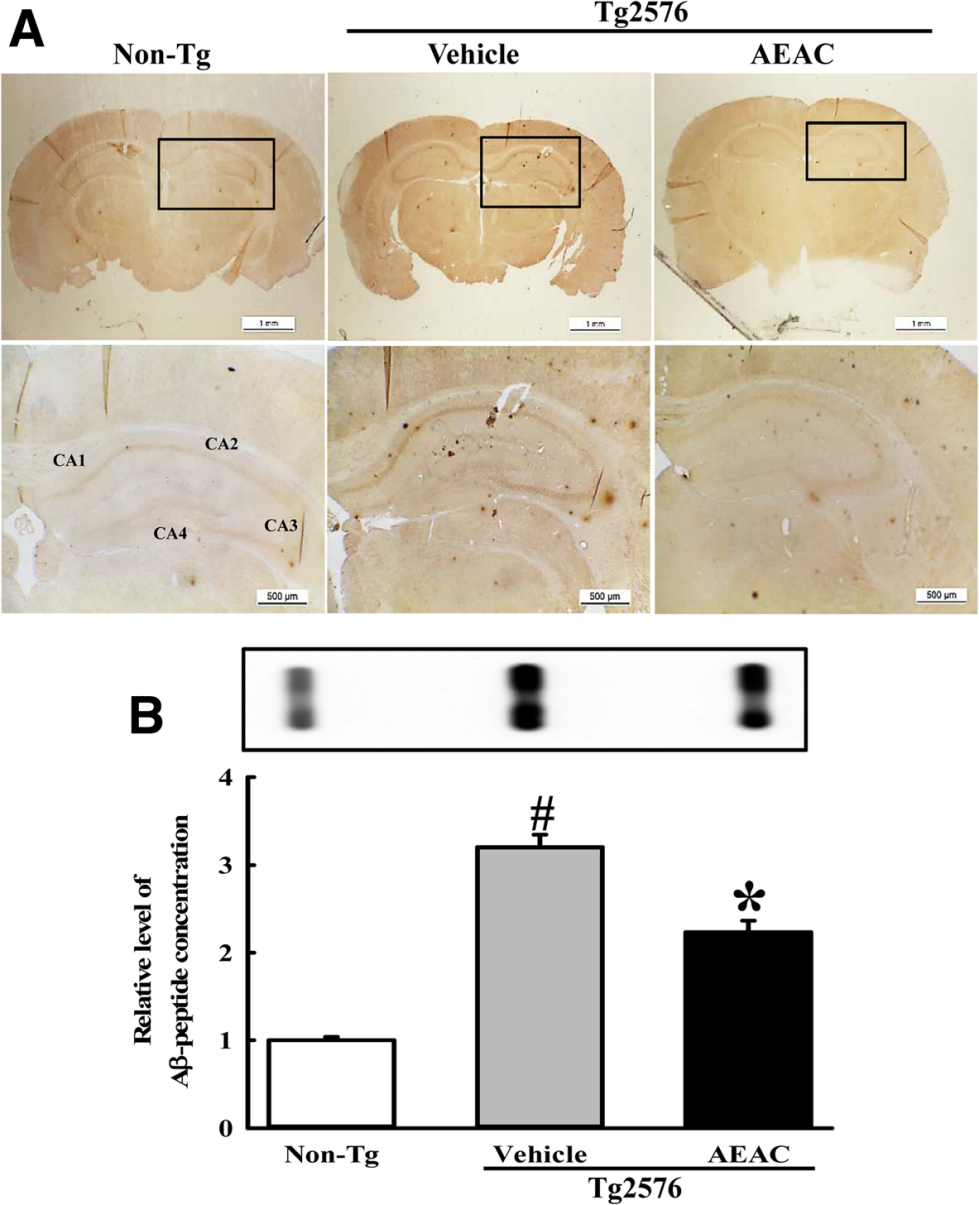
Deposition and concentration of Aβ-42 peptides. **a** Deposition of Aβ-42 peptides in brains of Tg2576 mice was detected by immunostaining using specific antibody. Whole brains including the hippocampus and cortex were observed at 10× (upper column), while detailed histological features of several regions of the hippocampus are shown in three rectangles at 400× (lower column). **b** Concentration of soluble Aβ-42 peptides in brains of Tg2576 mice was detected by dot blot assay. Data are reported as the means ± SD. ^#^, *P* < 0.05 compared to the Non-Tg group. *, *p* < 0.05 compared to the Vehicle treated Tg2576 group

To investigate whether the enhanced NGF level and anti-oxidant activity induced by AEAC treatment was accompanied by the reduction of Aβ-42 peptides production and accumulation, the concentration of Aβ-42 peptides and the expression level of γ-secretase key members was measured in the brain of Tg2576 mice after treatment for 4 weeks. Immunohistochemical analysis showed that the number of Aβ-42 stained cells was significantly lower in the hippocampus and cortex of AEAC treated Tg2576 mice than in Vehicle treated Tg2576 mice (Fig. 6a). A similar result was observed in the slot dot blot assay to detect the concentration of Aβ-42 peptides. A significant decrease of soluble Aβ-42 concentration was observed in the hippocampus of AEAC treated Tg2576 mice compared with Vehicle treated Tg2576 mice (Fig. 6b). These results suggest that AEAC treatment may contribute to a decrease in Aβ-42 concentration in the brain of Tg2576 mice.

**Fig. 7:**
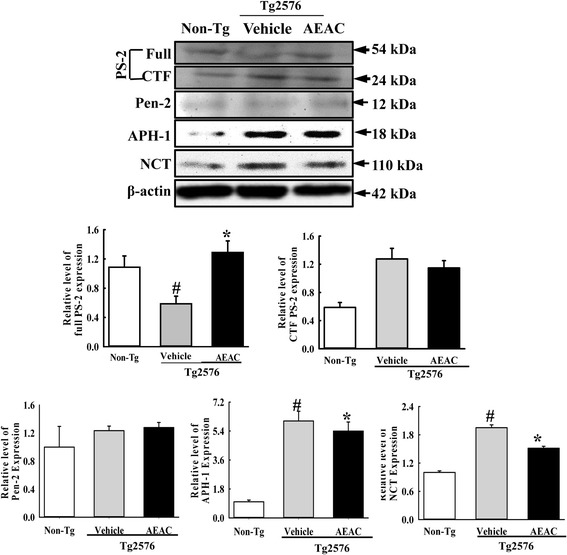
Expression of γ-secretase components in the brains of Tg2576 mice. Hippocampus regions were prepared from brain tissues of Vehicle and AEAC treated Tg2576 mice. Fifty micrograms of protein per sample was immunoblotted with antibody for each protein. Expression levels of the four γ-secretase components were measured with specific antibody and horseradish peroxidase conjugated goat anti-rabbit IgG, as described in the Methods section. The intensity of each protein was calculated using an imaging densitometer. Data are reported as the means ± SD from three replicates. ^#^, *P* < 0.05 compared to the Non-Tg group. *, *p* < 0.05 compared to the Vehicle treated Tg2576 group

Expression analysis of γ-secretase members revealed significant alterations in full PS-2, APH-1, and NCT after AEAC treatment. The expression levels of APH-1 and NCT were higher in the Vehicle treated Tg group than the Non-Tg group; however, they decreased significantly by 10.4% and 22.4% in the AEAC treated group although the expression of full PS-2 showed the reverse pattern in the same group (Fig. 7). These results indicate that the decrease in Aβ-42 peptides after AEAC treatment may be closely correlated with the suppression of PS-2, APH-1 and NCT expression.

### Effects of AEAC on the survival and function of neuronal cells in the brains of Tg2576 mice

**Fig. 8:**
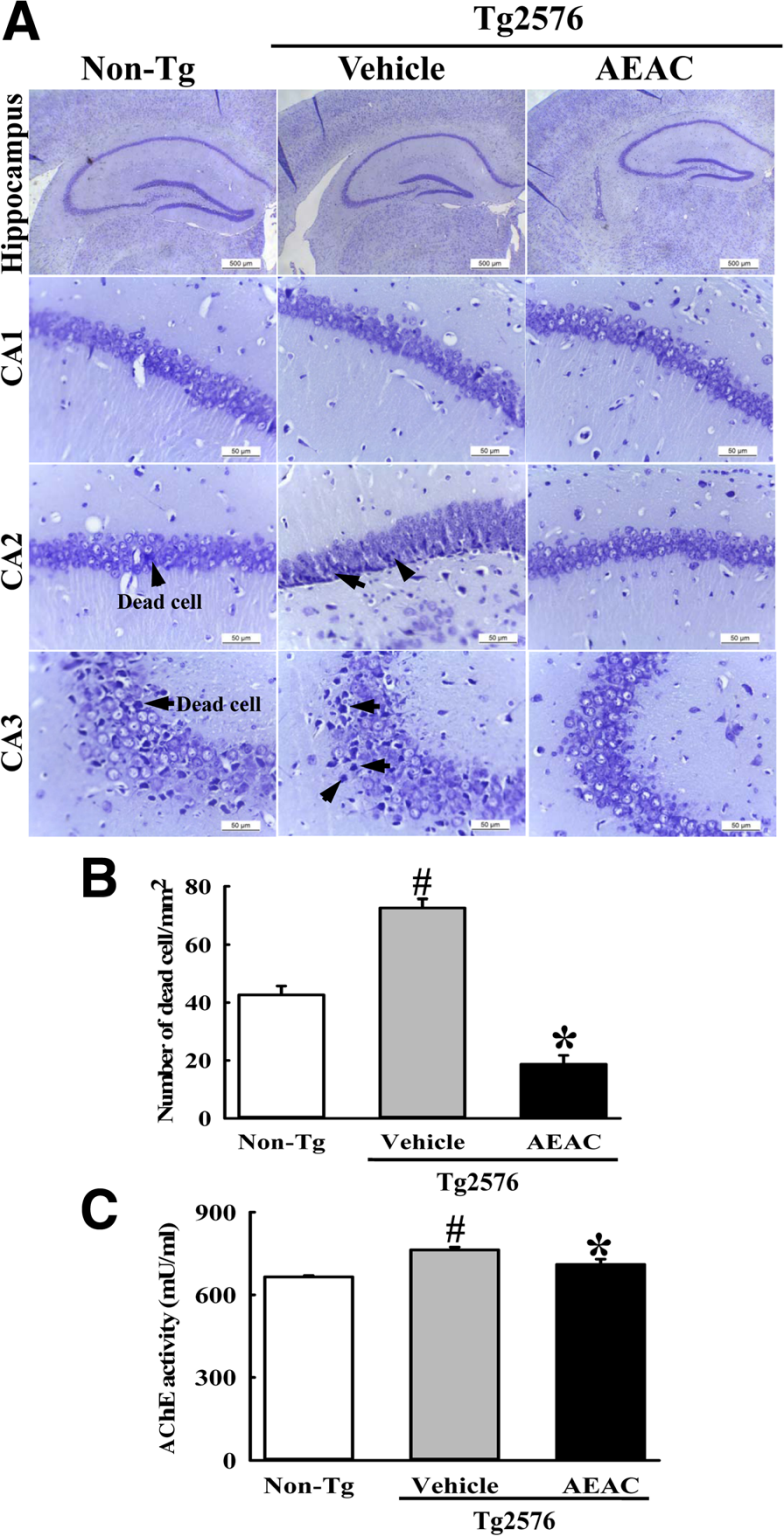
Survival and function in the hippocampus of Tg mice. **a** After AEAC administration for 4 weeks, brain tissues were collected from subset groups and histological changes were determined as described in the Methods. The slide sections of brain tissue were stained with Nissl and then observed at 400× magnification. **b** The total number of neuronal cells was calculated per 80 mm^2^. **c** AChE activity was measured using the homogenate of hippocampus tissue collected from two mice after AEAC treatment. Data shown are means ± SD (*n* = 5). ^#^, *P* < 0.05 compared with the Non - Tg group. *, *P* < 0.05 compared with the Vehicle treated Tg2576 group

To investigate whether AEAC treatment could affect the survival and function of neuronal cells, the number of dead neuronal cells and AChE activity were measured in the AEAC treated group. The number of dead neuronal cells was significantly higher in the granule cell layer of the dentate gyrus in the Vehicle treated Tg2576 group than the No treated group. However, their number decreased in the AEAC treated Tg2576 group relative to the Vehicle treated Tg2576 group (Fig. 8a and b). Furthermore, AChE activity showed the similar pattern, with the Vehicle treated Tg2576 group showing a higher level (14%) of AChE activity than the Non-Tg group. This level recovered significantly in the AEAC treated groups (Fig. 8c). Taken together, our findings indicate that AEAC treatment for 4 weeks can protect against the death and function of neuronal cells in the specific region of the brain in Tg2576 mice.

## Discussion

Multifunctional products exhibit the highest efficacy as the most effective strategy in the many potential experimental therapies for the treatment of neurodegenerative processes of AD because many cellular targets can be considered to prevent or retard the progression of AD to pathological condition [2]. As part of our research to develop these products, we are searching for novel natural products with multifunctions for therapeutic intervention at the pathological stage of AD. Among various targets, our study focused on stimulation of neurotropic secretion and suppression of oxidative stress. To accomplish this, AEAC was administrated to Tg2576 mice showing AD phenotypes for 4 weeks. The results of the present study are the first to suggest that AEAC can improve pathological conditions including Aβ-42 peptide deposition and neuronal cell dysfunction through enhanced NGF concentration and increased anti-oxidative activity in Tg2576 mice.

Neurotropic factors play a pivotal role in the mechanism underlying neuronal cell survival, differentiation of dendritic branching and dendritic spine morphology, synaptic plasticity and apoptosis of cells [10]. NGF, brain-derived neurotrophic factor (BDNF) and neurotrophins belonged to this neurotrophin family. Among these, NGF primarily contributes to maintenance of biological and morphological phenotype, survival and growth on mature basal forebrain cholinergic neurons [46–48]. Therefore, the regulation of NGF activity can provide an attractive therapeutic option to prevent cholinergic neuron degradation in AD [49]. However, pilot clinical trials were not investigated continuously because NGF cannot cross the brain blood barrier (BBB) and require intra-cerebroventricular infusion to arrive at target areas of the brain [50]. Therefore, most recent studies of NGF therapy have focused on the development of novel agents with the ability to increase the level of NGF secretion or enhance NGF activity. In this study, we investigated the therapeutic effects of AEAC on the pathological symptoms of the AD model through the upregulation of NGF secretion. Our results are the first to show that secretion of NGF and its metabolism in Tg2576 mice were effectively enhanced by administration of AEAC for 4 weeks.

Several studies have investigated the ability of compounds and natural products to stimulate NGF secretion in model animals for AD. Ginsenoside Rb1 from *Panax quinquefolium* L. increased the mRNA expression of NGF in the hippocampus [19], while spicatoside A isolated from *Liriope platyphylla* effectively induced enhancement of NGF in the serum of an animal model [51]. Moreover, increases in the NGF concentration of 65% and 163% were detected in the serum of NSE/hAPPsw Tg mice and Tg2576 mice treated with red *L. platyphylla* (RLP) manufactured by the steaming process [20, 21]. Furthermore, similar results with an 80–85% increase were observed in Tg2576 mice and TMT-injected ICR mice after the administration of fermented soybean products for several weeks [22, 23]. However, little is known about the use of AEAC treatment of neurodegenerative diseases or their direct effects on NGF secretion using animal models expressing AD pathological features. The results of our study are in agreement with the above reports, although there are few differences in the treatment condition and increase rate (79%). However, further studies are needed to understand what key factors determine the secretion of NGF and to verify the body distribution and duration time of AEAC after initial administration.

Meanwhile, the biological function of NGF transduce by interacting with two receptors, Trk and p75^NTR^ on the surface of cells. The action of these receptors associated with several signaling pathway including Akt and MAPK-induced signaling via Trk, and JNK, p53, and NF-kB signaling via p75^NTR^ [10]. The functional activity of this receptor should be investigated to verify the therapeutic effects of this novel NGF stimulator. In previous studies using the AD model, some differences in responses were detected in the expression of the Trk and p75^NTR^ signaling pathway. After treatment with RLP, downstream effectors of the NGF receptor signaling pathway, including TrkA and p75^NTR^ proteins, were suppressed in NSE/hAPPsw Tg mice and Tg2576 mice [20, 21]. However, the low phosphorylation levels of TrkA and Erk in the NGF receptor TrkA signaling pathway were rapidly recovered in Tg2576 mice treated with fermented soybean products, whereas the phosphorylation level of Akt was maintained at a constant level [23]. In our study, dramatic alterations in the p-Akt expression of the cortexes and hippocampuses of the Vehicle and AEAC treated group were detected among downstream members of the NGF receptor. However, their level was completely recovered to those of Non-Tg mice after AEAC treatment, while the level of p-ERK was slightly recovered (Figs. 4 and 5). Conversely, the decrease in p75^NTR^ was similar to the response of the RLP treated animal. We believe that this difference can be attributed to differences in the composition of treatment products, pathological severity caused by animal age and treatment period. Meanwhile, the recovery of p75NTR expression level did not agree with the phosphorylation level of JNK. These results suggested that the recovery signal of p75NTR induced by AEAC treatment may associated with other signals including NF-kB or p53 than JNK within downstream of p75NTR although additional studies will be needed.

The root of *A. cochinchinesis* has long been considered a therapeutic drug for human disease because of its anti-inflammatory, diuretic, antiseptic, antitussive, antibacterial, nervine, sialogoue, antipyretic, and stomachic effects [34]. At an early stage, these extracts are administered in combination with other herbs as medicine to treat lung disease, aging and immune response related diseases [36]. After treatment of these extracts and Compounds 2, 3 and 4 derived from them, secretion of pro-inflammatory cytokines such as TNF-α and NO concentration were significantly inhibited in LPS- and substance P-stimulated mouse astrocytes or microglial cells [52, 53]. Moreover, several indicators of skin inflammation progression including the degree of ectopic edema, ear thickness, cytokine secretion, and myeloperoxidase activity were greatly decreased by ethanol extract from *A. cochinchinesis* in a skin inflammation-induced mouse model treated with 12-O-tetradecanoyl-phorbol-13-acetate [54]. Furthermore, effective inhibition of TNFα–induced cytotoxicity [55], enhanced spleen index and SOD activity, and decreased malondialdehyde were observed in mice treated with the crude aqueous extract of *A. cochinchinensis*. Moreover, allergic asthma-associated airway remodeling and a decreased number of inflammatory cells were induced by treatment with standardized herbal formula PM014, which includes the roots of *A. cochinchinensis* [56]. However, the therapeutic effects of Aβ accumulated neurodegeneration in the brains of Tg2576 mice through stimulation of NGF secretion and anti-oxidant activity after *A. cochinchinensis* treatment have not been fully investigated, although some inflammatory effects of *A. cochinchinensis* in other organs have been studied. This is the first study to show a novel function and mechanism through which AEAC containing various compounds may relive the pathological symptoms of AD by enhancing NGF secretion and suppressing oxidative stress to reduce AD energetic failure. However, it should be noted that the present study was limited in that it used only one type of animal model for AD and single dose treated animals were only examined for up to 4 weeks. Additional multi-dose studies and model trials are necessary to clarify the therapeutic effects of AEAC and utilize this material to improve human health.

## Conclusions

In summary, the present study investigated the potential therapeutic action of AEAC for AD treatment, particularly its attenuation of Aβ deposition and neuronal dysfunction in Tg2576 mice. AEAC significantly decreased Aβ production and deposition and improved the survival and function of neuronal cells in the brains of Tg2576 mice through the stimulation of NGF activity and suppression of oxidative stress.

## Abbreviations

AChE: Acetyl choline esterase
AD: Alzheimer’s disease
AEAC: Aqueous extract of *A. cochinchinesis* root
CAT: Catalase
DPPH: 2, 2-diphenyl-1-picrylhydrazyl
ELISA: Enzyme-linked immunosorbent assay
NGF: Nerve growth factor
NO: Nitric oxide
NOS: Nitric oxide synthase
ROS: Reactive oxygen species
SOD: Superoxide dismutase

## References

[1] Ferrer I (2012). Defining Alzheimer as a common age-related neurodegenerative process not inevitably leading to dementia. Prog Neurobiol.

[2] Aso E, Ferrer I. Potential therapeutic strategies to prevent the progression of alzheimer to disease states. In Tech Open 2013; 10.5772/54783.

[3] Hardy J, Allsop D (1991). Amyloid deposition as the central event in the aetiology of Alzheimer's disease. Trends Pharmacol Sci.

[4] Mudher A, Lovestone S (2002). Alzheimer's disease–do tauists and baptists finally shake hands?. Trends Neurosci.

[5] De Strooper B, Vassar R, Golde T (2010). The secretases: enzymes with therapeutic potential in Alzheimer disease. Nat Rev Neurol.

[6] Morris M, Maeda S, Vossel K, Mucke L (2011). The many faces of tau. Neuron..

[7] Bonda DJ, Wang X, Perry G, Nunomura A, Tabaton M, Zhu X, Smith MA (2010). Oxidative stress in Alzheimer disease: a possibility for prevention. Neuropharmacology.

[8] McGeer PL, McGeer E, Rogers J, Sibley J (1990). Anti-inflammatory drugs and Alzheimer disease. Lancet.

[9] Ankarcrona M, Mangialasche F, Winblad B (2010). Rethinking alzheimer's disease therapy: are mitochondria the key?. J Alzheimers Dis.

[10] Kaplan DR, Miller FD (2000). Neurotrophin signal transduction in the nervous system. Curr Opin Neurobiol.

[11] Kapogiannis D, Mattson MP (2011). Disrupted energy metabolism and neuronal circuit dysfunction in cognitive impairment and Alzheimer's disease. Lancet Neurol.

[12] Francis PT, Ramírez MJ, Lai MK (2010). Neurochemical basis for symptomatic treatment of Alzheimer's disease. Neuropharmacology.

[13] Mufson EJ, Counts SE, Perez SE, Ginsberg SD (2008). Cholinergic system during the progression of Alzheimer's disease: therapeutic implications. Expert Rev Neurother.

[14] Bajda M, Guzior N, Ignasik M, Malawska B (2011). Multi-target-directed ligands in Alzheimer's disease treatment. Curr Med Chem.

[15] Numakawa T (2014). Possible protective action of neurotrophic factors and natural compounds against common neurodegenerative diseases. Neural Regen Res.

[16] Timm DE, de Haseth PL, Neet KE (1994). Comparative equilibrium denaturation studies of the neurotrophins: nerve growth factor, brain-derived neurotrophic factor, neurotrophin 3, and neurotrophin 4/5. Biochemistry.

[17] Chen ZA, Wang JL, Liu RT, Ren JP, Wen LQ, Chen XJ, Bian GX (2009). Liquiritin potentiate neurite outgrowth induced by nerve growth factor in PC12 cells. Cytotechnology.

[18] Yabe T, Tuchida H, Kiyohara H, Takeda T, Yamada H. Induction of NGF synthesis in astrocytes by onjisaponins of *Polygala tenuifolia*, constituents of kampo (Japanese herbal) medicine, Ninjin-yoei-to. Phytomedicine. 2003;10:106–14. 10.1078/094471103321659799 12725562

[19] Salim KN, McEwen BS, Chao HM (1997). Ginsenoside Rb1 regulates ChAT, NGF and trkA mRNA expression in the rat brain. Brain Res Mol Brain Res.

[20] Choi SI, Goo JS, Kim JE, Hwang IS, Lee HR, Lee YJ, Son HJ, Lee HS, Lee JS, Hwang DY. Effects of red *Liriope platyphylla* on NGF secretion ability., NGF receptor signaling pathway and r-secretase components in NSE/hAPPsw transgenic mice expressing alzheimer’s disease. Lab Anim Res. 2012;28:155–63. 10.5625/lar.2012.28.3.155 PMC3469843 23091515

[21] Choi SI, Go J, Kim JE, Lee YJ, Kwak MH, Jung YJ, Hwang DY. Precautionary effects of Red *Liriope platyphylla* on NGF secretion and Aβ42 deposition under the preclinical stage of Alzheimer's disease in Tg2576 mice. Lab Anim Res. 2013;29:212–20. 10.5625/lar.2013.29.4.212 PMC3879340 24396386

[22] Go J, Kim JE, Kwak MH, Koh EK, Song SH, Sung JE, Kim DS, Hong JT, Hwang DY. 2015. Neuroprotective effects of fermented soybean products (Cheonggukjang) manufactured by mixed culture of *Bacillus subtilis* MC31 and *Lactobacillus sakei* 383 on trimethyltin-induced cognitive defects mice. Nutr Neurosci. 2016;19:247–59. 10.1179/1476830515Y.0000000025 25923962

[23] Lee YJ, Kim JE, Kwak MH, Go J, Son HJ, Kim DS, Hwang DY (2013). In vitro and in vivo study of effects of fermented soybean product (chungkookjang) on NGF secretion ability and NGF receptor signaling pathway. Lab Anim Res.

[24] Koh EK, Yun WB, Kim JE, Song SH, Sung JE, Lee HA, Seo EJ, Jee SW, Bae CJ, Hwang DY (2016). Beneficial effect of diosgenin as a stimulator of NGF on the brain with neuronal damage induced by Aβ-42 accumulation and neurotoxicant injection. Lab Anim Res.

[25] Nabeshima T, Nitta A, Hasegawa T (1993). Impairment of learning and memory and the accessory symptom in aged rat as senile dementia model (3): Oral administration of propentofylline produces recovery of reduced NGF content in the brain of aged rats. Yakubutsu Seishin Kodo.

[26] Praticò D (2008). Evidence of oxidative stress in Alzheimer's disease brain and antioxidant therapy: lights and shadows. Ann NY Acad Sci.

[27] Terni B, Boada J, Portero-Otin M, Pamplona R, Ferrer I (2010). Mitochondrial ATP-synthase in the entorhinal cortex is a target of oxidative stress at stages I/II of Alzheimer's disease pathology. Brain Pathol.

[28] Richard T, Pawlus AD, Iglésias ML, Pedrot E, Waffo-Teguo P, Mérillon JM, Monti JP (2011). Neuroprotective properties of resveratrol and derivatives. Ann NY Acad Sci.

[29] Mancuso C, Bates TE, Butterfield DA, Calafato S, Cornelius C, De Lorenzo A, Dinkova Kostova AT, Calabrese V (2007). Natural antioxidants in Alzheimer's disease. Expert Opin Investig Drugs.

[30] Mandel SA, Amit T, Weinreb O, Reznichenko L, Youdim MB (2008). Simultaneous manipulation of multiple brain targets by green tea catechins: a potential neuroprotective strategy for Alzheimer and parkinson diseases. CNS Neurosci Ther.

[31] Spagnoli A, Lucca U, Menasce G, Bandera L, Cizza G (1991). Long-term acetyl-L-carnitine treatment in Alzheimer's disease. Neurology.

[32] Nishida Y, Yokota T, Takahashi T, Uchihara T, Jishage K, Mizusawa H (2006). Deletion of vitamin E enhances phenotype of Alzheimer disease model mouse. Biochem Biophys Res Commun.

[33] Harrison FE, Hosseini AH, McDonald MP, May JM (2009). Vitamin C reduces spatial learning deficits in middle-aged and very old APP/PSEN1 transgenic and wild-type mice. Pharmacol Biochem Behav.

[34] Jiangsu medical college. Dictionary of traditional chinese medicines. In: Zhong Yao Da Ci Dian. Shanghai: Shanghai Science and Technology Press; 1986. p. 2307-10.

[35] Xiong DS, Yu LX, Yan X, Guo C, Xiong Y. Effects of root and stem extracts of *Asparagus cochinchinensis* on biochemical indicators related to aging in the brain and liver of mice. Am J Chinese Med. 2011;39:719–26. 10.1142/S0192415X11009159 21721152

[36] Xiao PG. Modern chinese material medica, 2nd ed. Vol. 3. Beijing: Chemical Industry Press; 2002. p.150

[37] Lee HA, Kim JE, Song SH, Sung JE, Jung MG, Kim DS, Son HJ, Lee CY, Lee HS, Hwang DY (2016). Effects of an aqueous extract of asparagus cochinchinensis on the regulation of nerve growth factor in neuronal cells. J Life Sci.

[38] Lei L, Ou L, Yu X. The antioxidant effect of *Asparagus cochinchinensis* (Lour.) Merr. shoot in D-galactose induced mice aging model and in vitro. J Chin Med Assoc. 2016;79:205–11. 10.1016/j.jcma.2015.06.023 26935854

[39] Samad NB, Debnath T, Hasnat A, Pervin M, Kim DH, Jo JE, Park SR, Lim BO. Phenolic contents, antioxidant and anti-inflammatory activities of *Asparagus cochinchinensis* (Loureiro) Merrill. J Food Biochem. 2014;38:83–91.

[40] Son HL, Anh NP. Phytochemical composition, in vitro antioxidant and anticancer activities of quercetin from methanol extract of *Asparagus cochinchinensis* (LOUR.) Merr. Tuber. J Med Plant Res. 2013;7:3360–6.

[41] Singleton VL, Orthofer R, Lamuela-Raventos RM (1999). Analysis of total phenols and other oxidation substrates and antioxidants by means of folin-ciocalteu reagent. Methods Enzymol.

[42] Meda A, Lamien CE, Romito M, Millogo J, Nacoulma OG (2005). Determination of the total phenolic, flavonoid and proline contents in burkina fasan honey, as well as their radical scavenging activity. Food Chem.

[43] Hassan SM, Al Aqil AA, Attimarad M (2013). Determination of crude saponin and total flavonoids content in guar meal. Adv Med Plant Res.

[44] Oh H, Ko EK, Kim DH, Jang KK, Park SE, Lee HS, Kim YC. Secoiridoid glucosides with free radical scavenging activity from the leaves of *Syringa dilatata*. Phytother Res. 2003;17:417–9. 10.1002/ptr.1148 12722154

[45] Prajapati KD, Sharma SS, Roy N (2010). Upregulation of albumin expression in focal ischemic rat brain. Brain Res.

[46] Montero CN, Hefti F (1988). Rescue of lesioned septal cholinergic neurons by nerve growth factor: specificity and requirement for chronic treatment. J Neurosci.

[47] Allard S, Leon WC, Pakavathkumar P, Bruno MA, Ribeiro-da-Silva A, Cuello AC (2012). Impact of the NGF maturation and degradation pathway on the cortical cholinergic system phenotype. J Neurosci.

[48] Barres BA, Barde Y (2010). Neuronal and glial cell biology. Curr Opin Neurobiol.

[49] Cuello AC, Bruno MA, Allard S, Leon W, Iulita MF (2010). Cholinergic involvement in Alzheimer's disease. a link with NGF maturation and degradation. J Mol Neurosci.

[50] Eriksdotter JM, Nordberg A, Amberla K, Bäckman L, Ebendal T, Meyerson B, Olson L, Seiger Shigeta M, Theodorsson E, Viitanen M, Winblad B, Wahlund LO. Intracerebroventricular infusion of nerve growth factor in three patients with Alzheimer's disease. Dement. Geriatr Cogn Disord. 1998;9:246–57. 10.1159/000017069 9701676

[51] Kang TH, Kim SY. Comparison of nerve growth factor induction by butanol fraction of *Liriope platyphylla* and *Ophiopogon japonicas*. Kor J Pharmacogn. 2008;39:75–9.

[52] Kim HM, Lee EH, Lim TK, Jung JA, Lyu YS. Inhibitory effect of *Asparagus cochinchinensis* on tumor necrosis factor-alpha secretion from astrocytes. Int J Immunopharmacol. 1998;20:153–62. 10.1016/s0192-0561(98)00022-8 9730251

[53] Jian R, Zeng KW, Li J, Li N, Jiang Y, Tu P. Anti-neuroinflammatory constituents from *Asparagus cochinchinensis*. Fitoterapia. 2013;84:80–4. 10.1016/j.fitote.2012.10.011 23103295

[54] Shen Y, Xu Cl, Xuan WD, Li HL, Liu RH, Xu XK, Chen HS. A new furostanol saponin from *Asparagus cochinchinensis*. Arch Pharm Res. 2011;34:1587–91. 10.1007/s12272-011-1001-7 22076757

[55] Lee DY, Choo BK, Yoon TS, Cheon MS, Lee HW, Lee YA, Kim HK. Anti-inflammatory effects of *Asparagus cochinchinensis* extract in acute and chronic cutaneous inflammation. J Ethnopharmacol. 2009;121:28–34. 10.1016/j.jep.2008.07.006 18691647

[56] Jung KH, Choi HL, Park SJ, Lee GH, Kim MR, Min JK, Min BI, Bae HS (2014). The effects of the standardized herbal formula PM014 on pulmonary inflammation and airway responsiveness in a murine model of cockroach allergen-induced asthma. J Ethnopharmacol.

